# Banking of post-SMILE stromal lenticules for additive keratoplasty: A new challenge for eye banks?

**DOI:** 10.1007/s10792-024-03283-7

**Published:** 2024-08-25

**Authors:** Raluca Bievel-Radulescu, Stefano Ferrari, Moreno Piaia, Domitilla Mandatori, Assunta Pandolfi, Mario Nubile, Leonardo Mastropasqua, Horia Tudor Stanca, Diego Ponzin

**Affiliations:** 1 Fondazione Banca Degli Occhi del Veneto, Via Paccagnella, 11, 30174 Venice, Italy; 2 Department of Ophthalmology, “Carol Davila” University of Medicine and Pharmacy, 030167 Bucharest, Romania; 3 Department of Medicine and Aging Science, Ophthalmology Clinic, University G. D’Annunzio of Chieti-Pescara, 66100 Chieti, Italy; 4 StemTeCh Group, Department of Medical, Oral and Biotechnological Sciences, Center for Advanced Studies and Technology-CAST, University G. D’Annunzio of Chieti-Pescara, 66100 Chieti, Italy

**Keywords:** Small incision lenticule extraction, Eye banking, Post-SMILE stromal lenticules, Femtosecond lenticule extraction, Refractive surgery

## Abstract

**Purpose:**

ReLEx (Refractive Lenticule Extraction) Small Incision Lenticule Extraction (SMILE), the second generation of ReLEx Femtosecond Lenticule Extraction (FLEx), is a minimally invasive, flapless procedure designed to treat refractive errors such as myopia, hyperopia, presbyopia, and astigmatism. This review aims to provide a comprehensive overview of the methods for preserving SMILE-derived lenticules and discusses their potential future applications.

**Methods:**

A narrative literature review was conducted using PubMed, Scopus, and Web of Science databases, focusing on articles published up to January 2024 and available in English. The authors also evaluated the reference lists of the collected papers to identify any additional relevant research.

**Results:**

No standardized protocols currently exist for the storage or clinical application of SMILE-derived lenticules. However, these lenticules present a promising resource for therapeutic uses, particularly in addressing the shortage of donor corneal tissues. Their potential applications include inlay and overlay additive keratoplasty, as well as other ocular surface applications. Further research is needed to establish reliable protocols for their preservation and clinical use.

**Conclusion:**

SMILE-derived lenticules offer significant potential as an alternative to donor corneal tissues. Standardizing their storage and application methods could enhance their use in clinical settings.

## Introduction

**Fig. 1:**
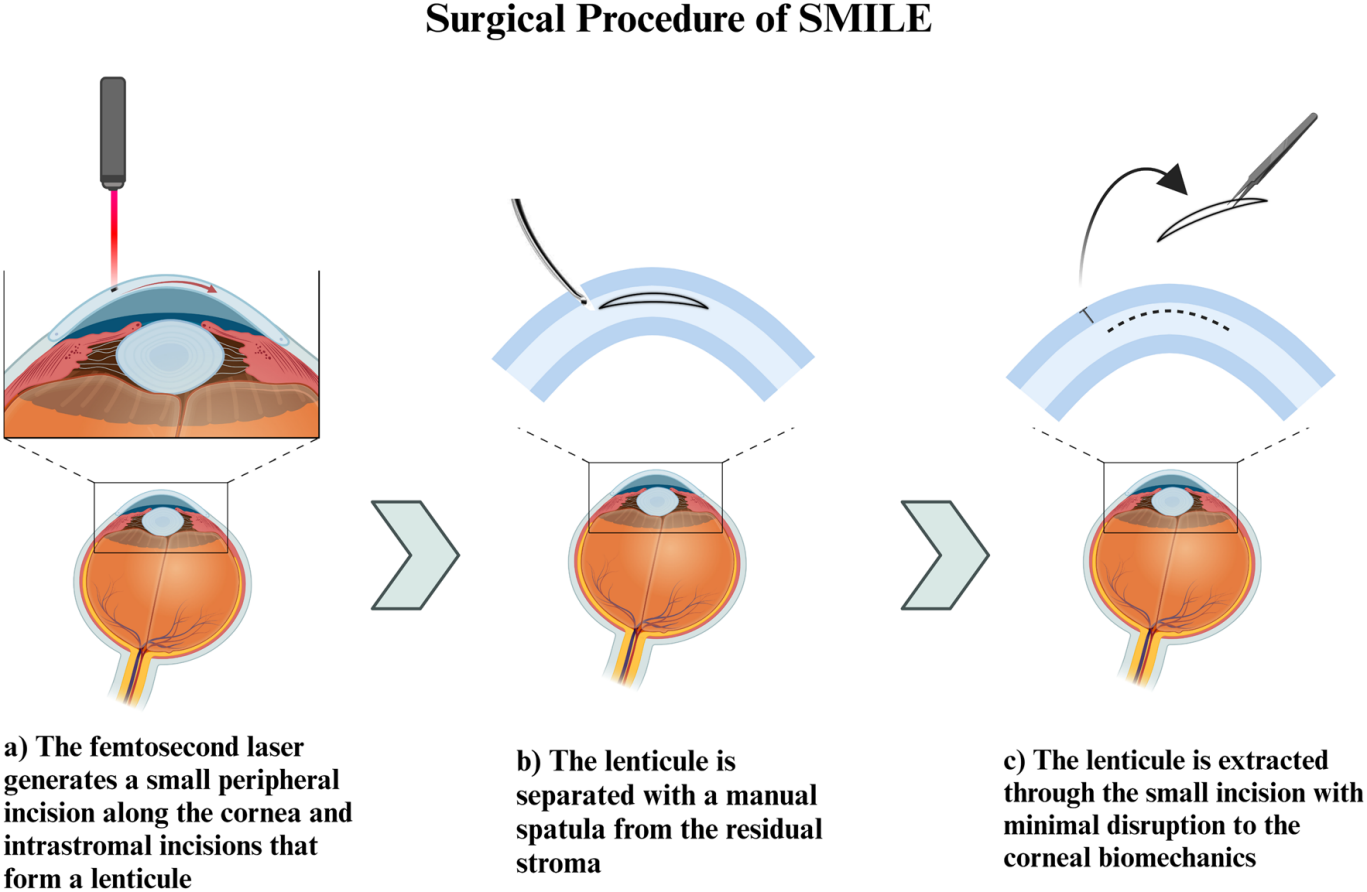
Illustration of the surgical procedure known as SMILE, showing the key steps in the corneal lenticule extraction process. Created with BioRender.com, accessed on 12 February 2024

The field of ophthalmology has been facing a continuous insufficiency of corneal tissues. A global survey conducted in 2016 assessed that over 12 million people worldwide are waiting for a corneal transplant, highlighting a mismatch of 1 cornea available for 70 needed [1]. The shortage of corneal grafts is explained by several factors. A primary reason that restricts donations underlies the limited number of healthcare infrastructures in many parts of the world, as well as cultural and social barriers [2, 3]. As a direct consequence, the transplantation waiting time is prolonged and the advancement of the disease and parallel impairment of the patient’s quality of life are inevitable. The intricate properties of the cornea to maintain its transparency and the uniqueness of its structure, contribute to the complexity and delicacy of its storage. The continuous growth and development of the field led the way for improved and more efficient storage conditions and tissue preservation methods [4]. Limited tissue wastage has become a primary objective as it is considered a factor that can be addressed to alleviate the shortage of corneal tissues. To achieve this goal, conservation methods have been refined, with the most common methods including hypothermic storage and organ culture [5, 6]. Previously, eye banks primarily supplied full-thickness corneas harvested from cadaveric donors for penetrating keratoplasty. However, research and innovation led by surgeons and scientists have driven advancements in surgical techniques for visual impairments, sparking a transformative revolution. The innovation of appliances and the expansion of ocular tissue types stored in eye banks allow for the possibility of a brighter future for patients. The progress of laser technology introduced the use of femtosecond lasers that can perform highly precise 3-dimensional cuts from corneal transplant tissues with the developments of laser technology [7]. The small incision lenticule extraction (SMILE) is a clinically available technology that involves the use of femtosecond lasers for the treatment of myopia, astigmatism, and hyperopia. As a result, an intrastromal lenticule is created and extracted through a small peripheral incision [8, 9] (Fig. 1). The corneal stromal lenticule is a thin and disc-shaped part of the cornea that is obtained during SMILE. SMILE-derived lenticules can be structurally customized and reshaped based on the purpose of their re-implantation [10]. Moreover, one of the characteristic advantages of stromal lenticule is its negligible immunogenicity due to the lack of corneal endothelium, which would take part in post-implant immune rejection [11]. In the past, the lenticule obtained as a by-product of the procedure was discarded and wasted since no surgical practices involved its employment. Currently, there has been a significant surge in the clinical application of post-SMILE stromal lenticules, driven by promising results in correcting a spectrum of refractive errors. These include hyperopia, presbyopia, keratoconus, corneal ectasia, corneal perforation, and various other corneal diseases [12]. Moreover, these stromal lenticules can serve a dual purpose by functioning as an ocular drug delivery system for a variety of active molecules [13]. In particular, the post-SMILE stromal lenticules have been used in additive keratoplasty with convincing clinical outcomes [14, 15]. The growing demand for SMILE in additive keratoplasty will require an excellent protocol for the transportation and storage of lenticules. Stromal lenticule banking can capitalize and leverage the excessive corneal material to diminish and even overcome the corneal tissue shortage and significantly improve the outcomes in corneal transplantation.

## Methods

In this narrative review, we explore the feasibility of banking post-SMILE stromal lenticules for future use in additive keratoplasty. We performed research on PubMed, Scopus and Web of Science on November 30, 2023, to identify relevant studies. We used keywords that comprised “SMILE” and “banking” in combination with “additive keratoplasty”, “stromal lenticules”, “storage methods”, and “clinical application”. We placed a restriction on articles published from any date to January 2024 and limited our selection to those written in English. Articles were independently screened for eligibility in two stages. The first stage by the titles and (where available) the abstracts of the search output. Second stage, full-text versions of papers selected by either reviewer were obtained for more detailed scrutiny. After using this tool, the authors reviewed and edited the content as needed and took responsibility for the publication’s content.

We did not perform a systematic search or assess the quality of the articles included in the current review, as this is a narrative review. This aspect and its implications are discussed in the revised manuscript.

## SMILE-derived lenticules for additive keratoplasty

**Fig. 2:**
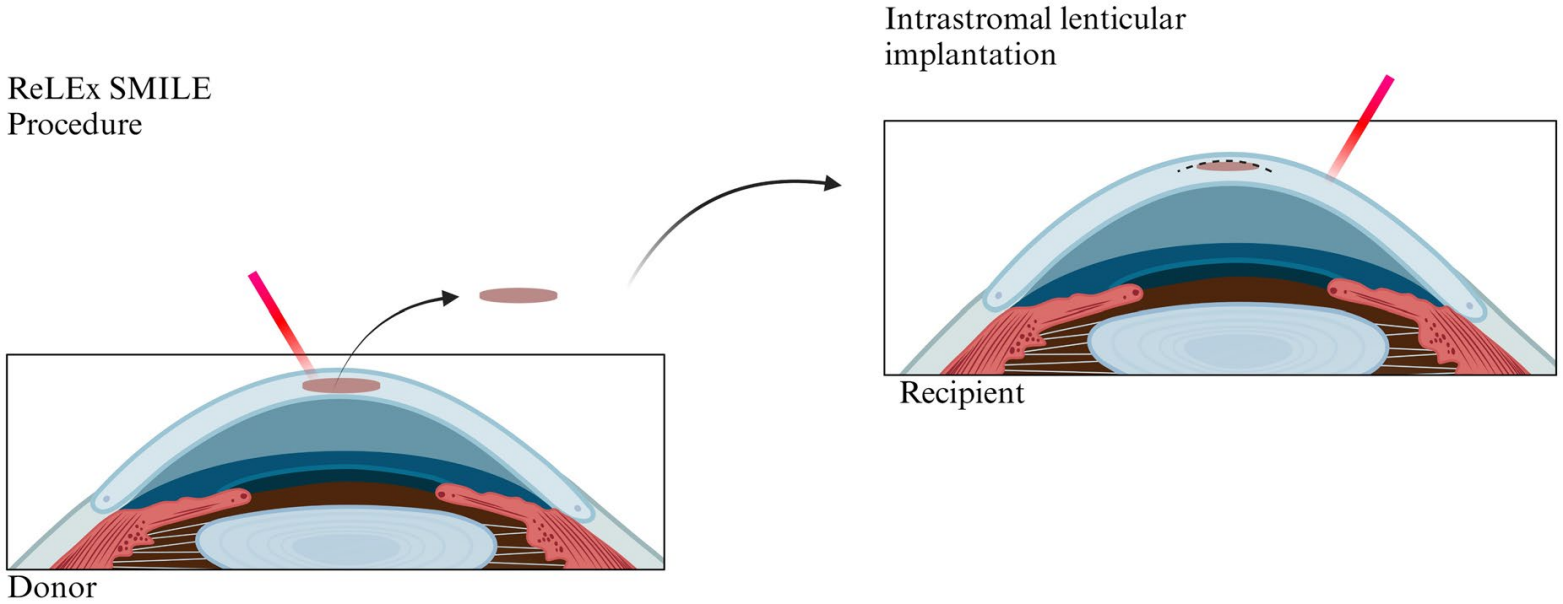
Utilization of the lenticule extracted during ReLEx SMILE surgery for intrastromal implantation. Created with BioRender.com, accessed on 12 February 2024

In 1966, Jose Ignacio Barraquer introduced a pioneering concept to the field of keratoplasty: the inception of corneal reshaping through tissue addition [16]. The term “keratophakia” originates from the Greek words “kerato” meaning “cornea” and “phakia” meaning “lens”. Keratophakia fundamentally constitutes a stromal tissue addition procedure, contributing to an augmentation of the recipient corneal volume (Fig. 2).

**Fig. 3:**
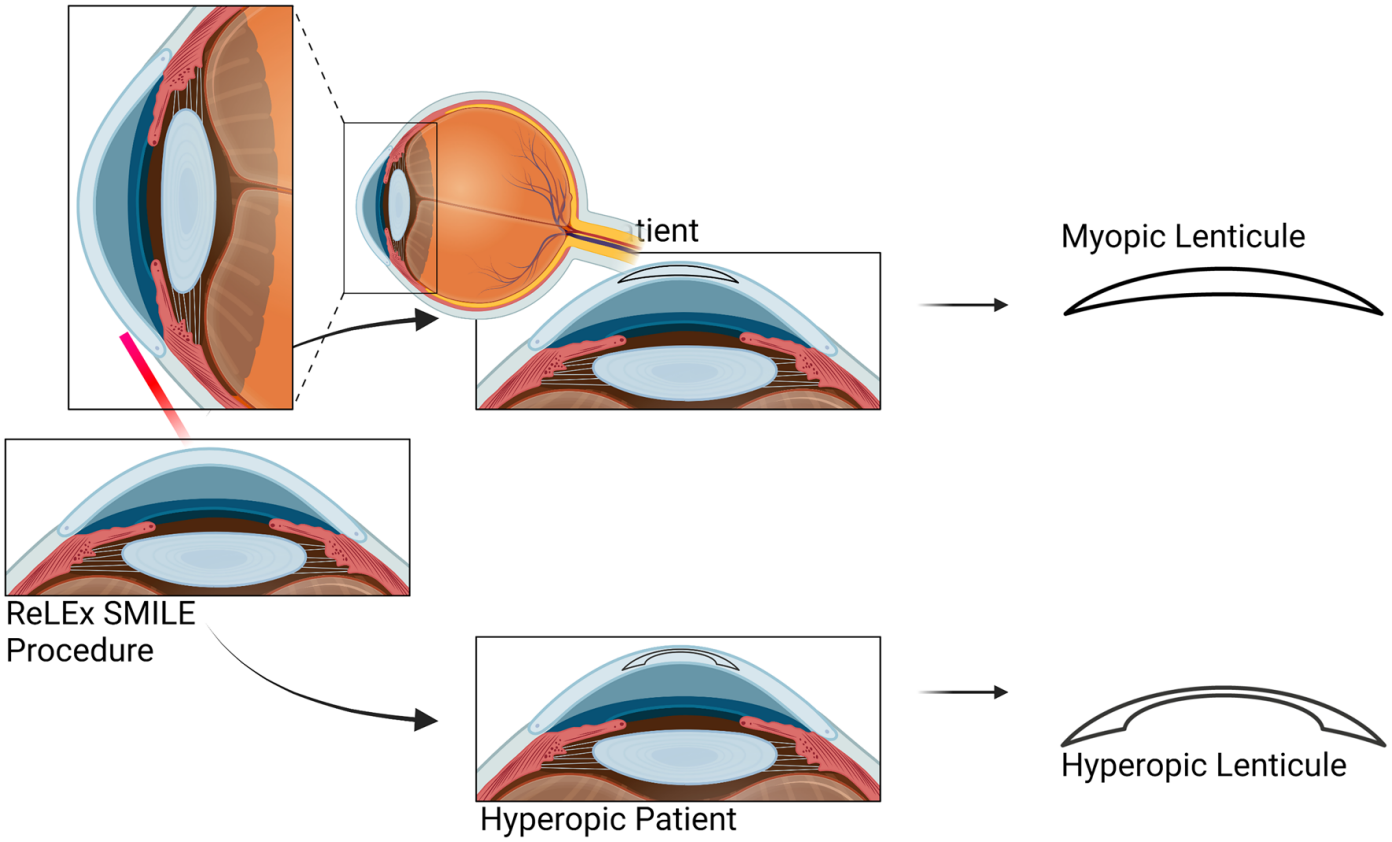
Disc-shaped pieces of tissue created during ReLEx SMILE. The lenticules for myopic correction have a convex form, thicker at the center and thinner at the edges. The lenticules for hyperopic correction have a concave form, thinner at the center and thicker at the edges. Created with BioRender.com, accessed on 12 February 2024

SMILE marked a revolutionary advancement in refractive surgery [17]. The procedure, characterized by its precision and reduced invasiveness, has become increasingly popular, offering patients a promising alternative to conventional method [18]. This is primarily attributed to its one-step procedure, facilitated by a femtosecond laser, where both the refractive lenticule and the flap are created simultaneously. This flap-free intrastromal laser-assisted technique involves the creation of a three-dimensional lenticule within the cornea, which is subsequently extracted through a small incision [19]. To date, more than 3.5 million SMILE procedures have been performed globally [20]. Disposing of these lenticules results in a significant loss of opportunities for potential applications. With the continually increasing number of SMILE surgeries, these lenticules represent a valuable resource that merits thorough study for potential repurposing in therapeutic applications. In the case of myopic treatment, the extracted lenticule exhibits a convex shape, indicating increased thickness at the center and reduced thickness at the periphery. In contrast, the hyperopic lenticule is characterized by a concave shape, as depicted in Fig. 3. Typically, the lenticule dimensions range from 6.0 to 6.5 mm, with its thickness determined by the corrected refractive power.

The introduction of femtosecond laser technology addressed the challenges associated with the precise creation of corneal pockets, a crucial step in lenticule implantation for stromal keratophakia. This technological advancement not only enabled more accurate and controlled surgical procedures but also promised enhanced clinical outcomes in stromal keratophakia. Referred to as the “all-in-one” femtosecond laser technique, this method enables the customization of implants, with a minimal incision size, ensuring a uniform depth and thickness with every procedure [21]. Lenticule implantation, essentially a form of selective lamellar keratoplasty, does retain a potential risk of rejection if the lenticule is not sourced from the recipient themselves. Nevertheless, the likelihood of rejection is anticipated to be lower when compared to full-thickness or lamellar corneal transplantation [22]. This is attributed to the reduced antigenic load of a lenticule composed solely of stromal tissue, in contrast to procedures like penetrating keratoplasty (PKP) or deep anterior lamellar keratoplasty (DALK), which involve the inclusion of more immunogenic epithelial and endothelial layers.

**Fig. 4:**
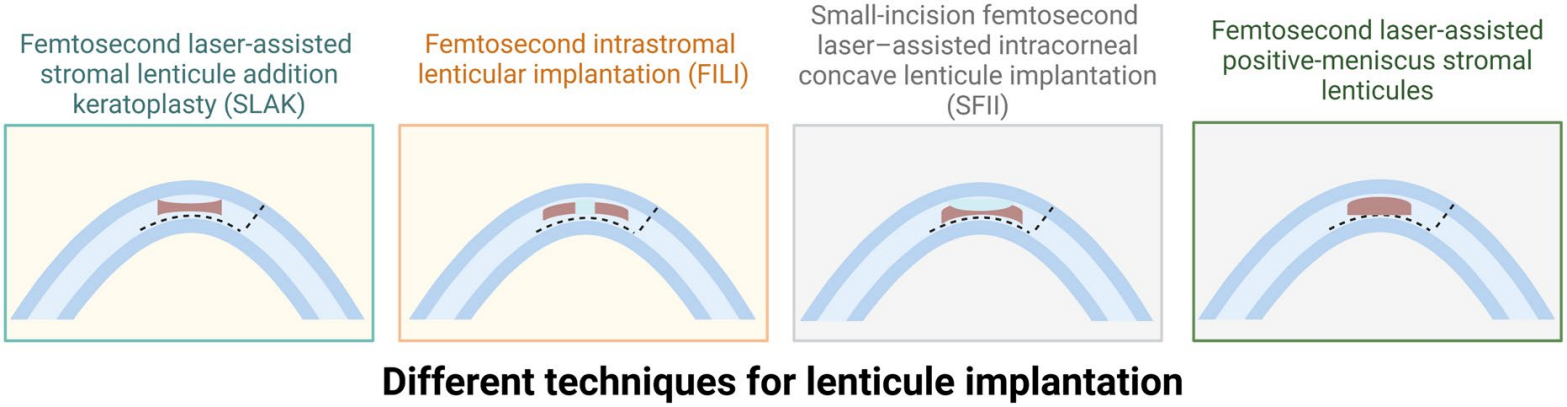
Illustration of various techniques for lenticule implantation. Created with BioRender.com, accessed on 12 February 2024

Various techniques for lenticule implantation have been developed, each offering unique approaches to address refractive and corneal disorders, as described below:

- Femtosecond laser-assisted stromal lenticule addition keratoplasty (SLAK), where the utilization of negative meniscus-shaped stromal lenticules induces a flattening of the cone while simultaneously augmenting corneal thickness [23].
- Femtosecond intrastromal lenticular implantation (FILI), where a donut-shaped tissue is utilized to reshape corneal tissue for the treatment of keratoconus [24]. Another application of FILI is for hyperopia [25].
- Small-incision femtosecond laser–assisted intracorneal concave lenticule implantation (SFII), involves the use of concave lenticules in the treatment of progressive keratoconus [26, 27].
- Femtosecond laser-assisted implantation of positive-meniscus stromal lenticules [28]. (Fig. 4)

These procedures are classified based on differences in the pocket depth, diameter, and thickness of the lenticule and represent a significant advancement in the field of refractive and corneal surgery. Following the creation of an intrastromal pocket using the femtosecond laser, the fresh or preserved lenticule may be implanted into a recipient cornea. This procedure serves to modify the corneal curvature.

## Banking of post-SMILE stromal lenticules

The promising advantages of post-SMILE stromal lenticules have opened the need to improve banking systems to reuse and cycle these tissues. In this vein, a standardized workflow from lenticule donation, testing, processing, and storage is pivotal to facilitating the distribution of lenticules for the patients. The stromal lenticules extraction is harmless for the donors, and their clinical application possesses safety perspectives for allogenic use since they have lower immunogenicity and risk of disease transmission. Currently, the stromal lenticules derived from refractive centers of surgery are considered waste products, although their use in clinical settings has been suggested as potentially profitable. The possibility of modifying the refraction of a cornea by inserting a refractive lenticule must be addressed both in vitro and in vivo, by examining the effect of storage and the consequent stromal swelling on the ultimate refractive power of the tissue. As most lenticules will be derived from the intermediate corneal stroma, which exhibits a rather regular structure, there is no need to mark the tissue to preserve orientation, although this must be confirmed experimentally. If confirmed, it will be possible to avoid the use of stromal dyes, which could persist for a long time once the lenticule is inserted.

Numerous hindrances complicate this process, including ethical and regulatory frameworks to be respected. For instance, current eye banking systems are not adequately funded for lenticule storage services for autologous transplantation, and all costs are borne by the donor. In addition, the possible allogeneic use of these tissues risks raising ethical and logistical problems, given that there is a lack of international policies for the procurement of banked stromal lenticules. An additional issue is the lack of international standards and accreditation policies in national and international registries that regulate the conservation and storage of lenticules [29]. Indeed, different countries adopt specific regulatory frameworks to regulate the clinical use of stromal lenticules, and the lack of international guidelines hinders the widespread distribution of these tissues. Therefore, it is crucial to establish specific regulations that regulate and coordinate the banking of post-SMILE stromal lenticules at an international level to facilitate the development of management and financing plans for their distribution.

## Storage methods for stromal lenticule

The evolution of bank systems among countries and the increased demand for lenticules for clinical and research applications have imposed the need to develop storage methods to optimize the transport of extracted lenticules for subsequent refractive surgery. Indeed, storage techniques are one of the main challenges in the use of SMILE-derived lenticules since they can largely affect the surgery outcomes in terms of visual acuity and complication risks. With regard to the standards for the collection of lenticules post-SMILE, inclusion and exclusion criteria for living donors will be similar to those adopted for the collection of corneoscleral tissues from deceased donors (compulsory serological tests, contraindications to donations, etc.). Guidelines from Competent Authorities will be strictly followed, but the risk/benefit ratio will have to be carefully assessed as transplantation of lenticules will be performed on subjects who might only suffer from refrative errors, not pathological disorders. However, similarly to the collection of amniotic membranes from living subjects, donor selection might be easier than with deceased donors, as more thorough anamnesistic controls and/or requests for additional serological analyses can also be performed.

The existing procedures for stromal lenticules conservation can be divided into short- and long-term storage based on their employment. Short-term storage methods mainly include fresh lenticules from living or cadaverous donors, while long-term storage methods include the use of tissue preservation procedures, typically cryopreservation or decellularization [30]. When it comes to lenticule implantation, particularly for refractive purposes, the activity of keratocytes does not significantly impact the corrective efficacy after implantation. This is supported by long-standing experience with epikeratophakia lenticules and anterior lamellar procedures, which indicates that a dehydrated, non-viable corneal lenticule can be safely utilized. These non-viable lenticules are slowly repopulated by host cells within two years following implantation, suggesting that the initial viability of keratocytes in the lenticule does not play a crucial role in the long-term clinical outcomes.

### Short-term storage methods

The optimal protocol to allow short-term storage of stromal lenticules from extraction to the storage facility centers has not been established. The principal hurdle concerns the heterogeneity in transport logistics systems between banking systems and the facilities for lenticule processing, which varies along the different nations. In developed countries, the transportation time for lenticules to specialized banking centers is short, while in countries without a proper banking system, the transfer of lenticules can be as long as 48 h. Accordingly, Liu et al. [31] propose 48 h as the maximum time of short-term storage for lenticule transportation. Hence, it is crucial to identify standardized transport media that allow the structural integrity of the tissue within 48 h of extraction [31]. To date, different medium solutions have been tested and demonstrated to preserve tissues for variable durations.

Among these, Liu and colleagues tested several media, including Dulbecco’s Modified Eagle’s Medium (DMEM) supplemented with 10% fetal bovine serum (FBS), phosphate-buffered saline (PBS), Optisol-GS, and pure glycerol. These compounds are effective in maintaining structural integrity, transparency, and low immunogenicity of lenticules under hypothermia (PBS, DMEM, and Optisol-GS) or at room temperature (glycerol) [31]. Notably, lenticules preserved in glycerol and subsequently cryopreserved at − 80 °C for four weeks showed inter-fibrillar spacing comparable to controls, probably because glycerol prevents the formation of ice crystals [31, 32]. The potential use of glycerol in preserving has been highlighted for its properties in generating tissue dehydration, which can maintain lenticular transparency and the biomechanical and molecular properties until subsequent tissue rehydration [33, 34]. Nonetheless, Liang et al. [35] stored fresh lenticules in glycerol, Optisol-GS, and allochroic silica gel desiccant at 4 °C until two weeks, showing extensive histological changes including tissue firregularity, cavitation bubbles, and edema of collagen fibers. Their study highlighted how only Optisol-GS maintained satisfactory standards in lenticule viability/integrity and collagen density, proposing Optisol-GS as the optimal storage medium for hypothermic preservation. In contrast, Optisol-GS can induce cytotoxic effects leading to apoptosis of stromal keratocytes, hindering its use for lenticular storage.

### Long-term storage methods

Nowadays, the use of fresh lenticules from living donors provides tissues with preserved tissue integrity, although they are exposed to the risk of infection or disease transmission [30]. The challenge at hand compels numerous countries to conduct serological tests, whereas corneas are usually made available within a few weeks. However, when dealing with data from a living donor, this process can extend up to 6 months. Hence, the usability of fresh lenticules can be unsuitable at times, necessitating the implementation of long-term preservation methods.

In this vein, cryopreservation is the most used method in clinical settings since it prevents pathogen transmission and ensures at least two years of storage [36, 37]. This storage method requires the use of cryoprotectant agents (i.e. dimethyl sulfoxide—DMSO) to prevent structural and functional changes in the stromal lenticules. The use of these compounds has been validated by several studies showing that DMSO alone or in combination with FBS maintained the fibrillar architecture, clarity, and sphericity of stromal lenticules [36–38]. Notably, numerous pieces of evidence report that cryopreservation maintains most of the metabolic activities and cell viability in the stromal lenticules [29, 36–38] despite observing an increase in apoptotic cells [36]. Riau et al. [39] investigated the outcomes of re-implanted cryopreserved refractive lenticules after eight weeks, reporting the absence of myofibroblasts or abnormal collagen type I expression within the cornea. They also reported a significant reduction of fibronectin and tenascin expression in the following eight weeks, suggesting the potential utility of cryopreservation [39]. Conversely, studies on rabbit models of monocular endokeratophakia showed a rise in apoptotic keratocytes and in the deposition of fibronectin and tenascin six months after re-implantation although the corneas displayed optimal clarity and refraction [40]. Besides, previous findings reported that stromal keratophakia using cryo-preserved lenticules leads to impaired stromal clarity due to the damage of stromal lamellar architecture and the development of interface scarring and edema, as well as in the absence of a proper innervation and keratocyte re-population [41, 42]. In addition, Bandeira et al. [43] observed neuron degeneration and decreased excitatory neurotransmitter-induced calcium levels, suggesting that cryopreservation may negatively impact nerve regeneration of post-transplanted stromal lenticules. Overall, cryopreservation is a safe procedure for long-term storage of stromal lenticules, which preserves most of the biological features of the cells, even if it can damage the stromal milieu and the clarity of the tissue.

Another storage method for preserving stromal lenticules is decellularization, which consists of removing the cellular components from the tissue through chemical or physical processes [44]. This technique provides acellular scaffolds with low immunogenicity, reducing the risk of host immune rejection in the implanted lenticule [11] and allowing the use of allogenic transplants [45, 46]. The decellularized ECM scaffolds possess a well-preserved composition of glycosaminoglycans and structural proteins, including fibronectin and collagen that mimic the normal stromal microenvironment and its biomechanical properties [29]. Conversely, in Yu et al. it has been shown that decellularized lenticules crosslinked with 2% Triton X-100 and 1% sodium dodecyl sulfate (SDS) lead to reduced transmittance and glycosaminoglycan levels, with a higher collagen fibril spacing [47]. In the same way, Shang and colleagues reported that decellularized post-SMILE stromal lenticules using 0.1% SDS treatment generate disorganized collagen fibers and a lower transmittance and Young’s modulus [11]. These pieces of information corroborate the idea that decellularization may improve immunogenicity despite an increase in tissue stiffness, which can impair the biomechanical properties of the lenticule.

## Clinical applications of corneal stromal lenticules

### Keratoconus and corneal ectasia

Keratoconus is one of the most common primary ectasias, a bilateral and asymmetric disease that results in progressive thinning of the cornea. This can lead to irregular astigmatism and decreased visual acuity [48]. The global prevalence was estimated to be 1.38 per 1000 population, which typically occurred between 20 and 30 years old [48, 49]. A range of treatment options exists for keratoconus, including eyeglasses and contact lenses in the early stages, cross-linking to stabilize disease progression, intrastromal corneal ring segments for reducing refractive errors or flattening the cornea, and more invasive procedures like penetrating keratoplasty and deep anterior lamellar keratoplasty. Recent advancements in the field encompass innovative techniques such as Bowman’s layer transplantation, stromal keratophakia, and stromal regeneration [50].

In this chapter, our central focus will be on stromal keratophakia for keratoconus, which involves the insertion of a lamella from the donor cornea into a pocket sculpted within the host’s stroma. (Table 1).

**Table 1:** Comparative findings in various types of additive keratoplasty for keratoconus

Author	Year	Country	Study design	Number of eyes	Mean age	Type of treatment	Pocket depth	Form of the lenticule	Follow-up (months)	Findings
Jafarinasab et al. [22]	2021	India	Prospective	5	29	Femtosecond Laser-assisted Allogenic Stromal Keratoplasty With or Without Excimer Laser Donor Keratomileusis	360 µm	concave	28.4	A regularization of the anterior surface of the cornea was achieved, along with a correction of refractive errors
Pedrotti et al. [28]	2021	Italy	Trial	34	/	Femtosecond laser-assisted stromal lenticule addition	200 µm above the endothelium	biconvex	/	The corneal thickness was improved, and there was a satisfactory restoration of the posterior surface
Pedrotti et al. [54]	2023	Italy	Prospective	15	47	Femtosecond intrastromal lenticular implantation	100 µm above the endothelium	positive meniscus lamella	12	MS-SLAK appears to be effective in achieving corneal surface regularization
Ganesh et al. [24]	2015	India	Prospective	6	19.5	FILI accelerated collagen cross-linking	100 µm	donut-shape	6	A clinical improvement in best corrected visual acuity was observed, along with a flattening of mean keratometry in both the 3-mm and 5-mm zones, accompanied by a reduction in higher-order aberrations
Mastropasqua et al. [23]	2018	Italy	/	10	/	Stromal lenticule addition keratoplasty	/	negative meniscus lamella	6	The negative meniscus-shaped lenticule addition induced corneal flattening, resulting in an improvement in visual acuity
Jadidi et al. [52]	2018	Iran	Prospective	4	27.25	FAISCG	250 µm	central keratoconus- circular shape; inferior keratoconus- crescent shape; in asymmetric bow-tie keratoconus- round shape	12	They successfully achieved a reduction in Kmax, accompanied by improvements in both best corrected visual acuity and spherical equivalent
Jin et al. [26]	2019	China	Retrospective	11	22.18	Small-incision femtosecond laser–assisted intracorneal concave lenticule implantation	160 µm	concave	24	The SFII group demonstrated enhanced corneal regularization, leading to superior visual acuity outcomes within the same group
Doroodgar et al. [53]	2020	Iran	Prospective	22	/	Femtosecond laser with customized Stromal Lenticule Implantation	/	simple necklace or necklace-with-ring shape	/	An improvement was achieved in vision, topography, and refraction

MS-SLAK, meniscus-shaped stromal lenticule addition keratoplasty; FILI, femtosecond intrastromal lenticular implantation; FAISCG, femtosecond-assisted intrastromal corneal graft; Kmax, maximum curvature power; SFII, small-incision femtosecond laser-assisted intracorneal concave lenticule implantation

To our knowledge, Ganesh et al. [24] employed for the first time cryopreserved corneal lenticules for the treatment of keratoconus following ReLEx SMILE, utilizing a donut-shaped configuration as donor material for the femtosecond intrastromal lenticular implantation technique (FILI) combined with crosslinking. FILI involves integrating natural corneal tissue, inducing localized elevation in the midperiphery and relative flattening in the center. They created a pocket for lenticule insertion within the patient’s cornea at a depth of 100 μm, with a diameter ranging from 7.0 to 8.0 mm. They obtained a significant reduction in the spherical aberration. This reduction was accompanied by a simultaneous decrease in both higher-order and coma aberrations across all eyes. Both uncorrected and best-corrected visual acuities exhibited improvement and were consistently maintained throughout the six months follow-up period. The effects of a novel femtosecond laser-assisted procedure for the patients with advanced keratoconus, termed stromal lenticule addition keratoplasty, were investigated in-vivo by Mastropasqua et al. They utilized negative meniscus-shaped stromal lenticules produced from corneoscleral eye bank buttons using a refractive lenticule extraction procedure. After a 6-month follow-up, both uncorrected and corrected distance visual acuity showed improvement. Corneal topography revealed a flattening of the cone, while anterior segment optical coherence tomography indicated a significant increase in corneal thickness [23]. In another study conducted by Mastropasqua et al., the effects of utilizing the same femtosecond laser-assisted stromal lenticule addition keratoplasty technique with negative meniscus-shaped stromal lenticules were investigated using confocal microscopy. At 12-month follow-up, the researchers reported a temporary decrease in nerve plexus density and minor inflammatory reactions, which significantly diminished within the initial month. The study also noted similarities in donor-recipient interface reflectivity with other femtosecond laser refractive procedures and observed no stromal opacification or rejection [51]. A recent technique involving a modified allogenic intrastromal lenticule implantation combined with corneal crosslinking for the treatment of advanced keratoconus has been described. In this approach, three out of five recipient eyes underwent excimer laser treatment for refractive error correction. The combination of these techniques, femtosecond laser-assisted allogenic stromal keratoplasty with excimer laser-assisted donor keratomileusis, showed superior refractive and keratometric outcomes [22].

Jadidi and colleagues utilized femtosecond laser technology to craft a tailored corneal lenticule with precise dimensions and shape, along with an intra-stromal pocket, in keratoconus patients [52]. The lenticule shape was determined based on the type of keratoconus: a circular shape for central keratoconus, a crescent shape for inferior keratoconus, and a round shape with a size adjusted for mesopic pupil size in asymmetric bowtie keratoconus. The entry point for the intracorneal pocket in the recipient eye was strategically positioned on the steepest corneal topographic axis using the femtosecond surgical laser, with the pocket depth set at 250 µm of the corneal thickness at the incision site. The utilization of the femtosecond-assisted intrastromal corneal graft technique resulted in a noteworthy transformation of the corneal surface geometry, leading to a general regularization of the corneal shape. According to their report, all patients demonstrated a significant improvement in corrected distance visual acuity [52]. Doroodgar et al. successfully implanted customized corneal stromal lenticules, using a simple necklace or necklace-with-ring shape based on the corneal thickness and topography configuration of the implanted keratoconic eyes. Corneal thickness increased by 100.4 µm at the thinnest point. No inflammatory features were observed due to the implanted fresh lenticules, and corrected distance visual acuity (CDVA) improved from 0.70 to 0.49 logMAR (P = 0.001). Additionally, keratometry decreased from 54.68 ± 2.77 to 51.95 ± 2.21 diopters (P = 0.006) [53].

An alternative technique employed for advanced keratoconus in cases where there is intolerance to contact lenses is meniscus-shaped stromal lenticule addition leratoplasty (MS-SLAK). In this procedure, a positive meniscus lamella, thicker in the center than in the periphery, is implanted into an intrastromal pocket sculpted 100 μm above the endothelium. The lenticule radius is determined by the greatest distance between the keratoconus apex and its periphery, encompassing the entire ectatic area and within 2 mm from the limbus. While an increase in corneal thickness was anticipated post MS-SLAK, a noteworthy finding was the regularization of the anterior corneal surface. The procedure exhibited significant improvements in topographic symmetry indices, a reduction in coma aberration, and a decrease in higher-order aberrations. These outcomes evidence the MS-SLAK efficacy in promoting corneal symmetry and the restoration of contact lens wearing tolerance [54].

The effectiveness of small-incision femtosecond laser-assisted intracorneal concave lenticule implantation (SFII) and penetrating keratoplasty was assessed in individuals with progressive keratoconus [26]. Both surgical interventions demonstrated enhanced visual acuity and maintained stable corneal curvature and thickness at the center 24 months postoperatively. Notably, the SFII group exhibited more evident corneal biomechanical changes. In contrast, penetrating keratoplasty was associated with the presence of more dendritic and inflammatory cells. This outcome suggests that the SFII procedure is minimally invasive, safe, and effective in treating progressive keratoconus, reducing the risk of graft-versus-host disease [26]

### Hyperopia

Hyperopia is a prevalent refractive condition in both children and adults, characterized by the eye’s tendency to focus parallel light rays from infinity behind the neurosensory retina, particularly when accommodation is at rest, following refraction through the ocular media. In contrast to the excellent clinical outcomes observed in terms of stability, predictability, safety, and efficacy for myopia correction, the results for hyperopia correction were comparatively less impressive in terms of stability, with high regression rate post-LASIK [55, 56]. Utilizing femtosecond laser-assisted stromal keratophakia, the implantation of a convex-shaped lenticule from SMILE surgery, originally designed for myopia correction, presents a promising avenue for hyperopia treatment. The effectiveness of this approach has been demonstrated in both humans and primates [57].

The pioneering implantation of a lenticule in humans was initially reported by Pradhan et al. [58]. In this procedure, an allogeneic lenticule obtained through SMILE from a myopic donor was successfully implanted for the correction of high hypermetropia in a young aphakic woman. By implanting a convex-shaped lenticule harvested from a myopic-SMILE procedure inside a stromal pocket, the anterior corneal curvature can theoretically be reshaped to be steeper. Ganesh et al. [36] explored the feasibility of correcting hyperopia using cryopreserved lenticules collected after ReLEx SMILE. The lenticules, stored in liquid nitrogen for an average of 96 days, were then inserted into 9 patients with hyperopia. It was utilized a femtosecond laser intrastromal lenticular implantation (FILI) for this procedure. All eyes experienced central corneal steepening. Importantly, higher order aberrations did not exhibit a significant increase postoperatively. These findings suggest the potential effectiveness of using cryopreserved lenticules for hyperopia correction through FILI, with maintained corneal structure and minimal impact on higher order aberrations. Previous work by the Tissue Engineering and Stem Cell Group at the Singapore Eye Research Institute had already demonstrated the viability of corneal lenticules extracted post-ReLEx SMILE, showing well-preserved and well-aligned collagen structures one month after cryopreservation [38].

Zhang et al. [59] conducted the first study on the outcomes of patients with astigmatic hyperopia treated with SMILE combined with an intrastromal lenticule inlay. In this study, they performed a SMILE procedure with -0.50D myopia and astigmatism in the hyperopic astigmatism eye. Simultaneously, patients with a myopic refractive error corresponding to the absolute value of residual hyperopia were scheduled for a routine SMILE procedure. The study demonstrated that uncorrected near visual acuity improved significantly from 0.49 to 0.08, and spherical equivalent improved from + 7.42D to − 0.75D 1 year after surgery compared to preoperative values. These findings indicate that SMILE with the allogeneic lenticule inlay was effective in improving visual outcomes for patients with astigmatic hyperopia.

### Presbyopia

Presbyopia is a typical age-related condition characterized by the progressive reduction in eye focusing range. This leads to a scenario where, even with correction for distance vision, the point of focus becomes insufficient for clear vision at close distances, impacting an individual’s ability to meet their visual needs [60]. Presbyopia, typically manifesting after the age of 40, has evolved into a global public health concern with the aging population. Current approaches for addressing presbyopia encompass various strategies such as spectacle lenses (including monovision, bifocal, trifocal, or progressive addition lenses), contact lenses (including monovision, multifocal, or modified monovision), surgical interventions (intraocular lenses, corneal inlays, or laser refractive surgery), and pharmaceutical treatments [61]. Some published reports have suggested that lenticules obtained from SMILE surgery could be explored as a potential treatment option for presbyopia. A novel technique, termed PrEsbyopic Allogenic Refractive Lenticule (PEARL) inlay, involves the use of an allogenic corneal inlay created from a SMILE. In this approach, a specified thickness post-SMILE lenticule (mean: 61.5 µm) is trephined at the center to a 1-mm diameter and implanted in the cornea beneath a femtosecond laser-created cap of 120 µm depth. Following the procedure, the uncorrected near visual acuity at 33 cm in the operated eye improved from J8 to J2. This is done in the nondominant eye of presbyopic patients. The preliminary study showed the safety and efficacy of the PEARL corneal inlay for presbyopic correction [62].

### Corneal perforation

Corneal ulcers and perforations are frequently responsible for a substantial reduction in visual acuity and, in some cases, can lead to vision loss. This condition constitutes a potentially vision-threatening ocular emergency [63]. The origins of these issues can be infectious, traumatic, or autoimmune, and in some cases, the exact cause of the corneal tissue disintegration remains unclear. The progression typically begins with a partial-thickness defect in the corneal epithelium, advancing to stromal invasion and, ultimately, resulting in a full-thickness perforation. Urgent intervention is imperative to cover the defect, restore eyeball integrity, and prevent intraocular tissue infection [64]. Current therapeutic approaches for corneal ulcers and perforations encompass various temporary treatments, such as amniotic membrane transplantation, tissue glue, conjunctival flaps or corneal transplant [65–68]. The application of stromal lenticules extracted through femtosecond laser SMILE surgery was investigated as a surgical adjuvant for sealing corneal perforations.

Lenticules with a central thickness of 100 μm or more were affixed over corneal perforation sites using 10–0 nylon interrupted stitches along with an overlying single layer of amniotic membrane. Throughout the one-year follow-up period, no indications of re-perforation or infections were observed in any patient. Additionally, three out of seven patients experienced an improvement in best-corrected visual acuity [69]. Another study had provided confirmation that corneal lenticules can serve as a safe and effective surgical alternative for closing corneal perforations. This approach presents a potential clinical application as a relatively straightforward and cost-effective temporary measure to enhance the condition of the cornea, paving the way for subsequent definitive interventions. In this study, the same technique was applied, with corneal lenticules being affixed over corneal perforation sites using 10–0 nylon interrupted stitches [70]. Corneal stromal lenticules obtained through femtosecond laser lenticule extraction have demonstrated applicability in the treatment of sizable corneal perforations exceeding 3 mm. These lenticules, preserved in glycerol at − 80 °C, feature a diameter ranging from 6.0 to 6.5 mm and a central thickness of 300 to 400 µm. Employed as an emergent therapy, their utilization underscores the significance of having readily available lenticules in countries facing a scarcity of cornea donors. This approach not only addresses the immediate need for treatment but also highlights the practicality of utilizing lenticules as a valuable resource in regions with limited access to corneal grafts [71].

A clinical study conducted on 22 patients experiencing corneal ulcers and perforations demonstrated the safety and efficacy of tectonic keratoplasty with femtosecond laser intrastromal lenticule (TEKIL). Following the TEKIL procedure, complete integrity was achieved globally in all cases. Importantly, no instances of immune rejection or perforation were detected, emphasizing the positive outcomes and potential benefits of TEKIL as a viable treatment approach for corneal ulcerations and perforations [72].

Tectonic keratoplasty employing SMILE has proven to be a viable option even in the pediatric population with blepharokeratoconjunctivitis. In both cases, viscoelastic material was introduced into the anterior chamber from the site opposite the perforation to uphold the anterior chamber depth. The procedure resulted in healing of the corneal perforation, maintenance of globe integrity, and favorable visual outcomes. Upon follow-up examinations, there were no indications of graft melting, graft rejection, corneal neovascularization, or infection in the patients [73].

In an *ex-vivo* study, it was tested the safety and efficacy of stromal lenticules obtained from SMILE compared with amniotic membrane graft for the treatment of perforated corneal ulcers. The study included 40 eyes with medium-sized corneal perforations. Adequate healing of corneal perforations was observed within a few weeks without significant complications. The stromal lenticule obtained from SMILE surgery demonstrated a tendency to be safer with faster healing compared to an amniotic membrane graft augmented with platelet-rich plasma [74].

## Conclusions

Existing evidence suggests that SMILE has ameliorated the management of multiple corneal disorders and holds promise for significantly improving refractive surgery. The low invasiveness and the high precision of SMILE may be a valid alternative to conventional clinical therapies, which typically include several risks and side effects for the patients. In this vein, novel therapeutic approaches using post-stromal SMILE lenticules can be developed to comply with the continuous insufficiency of corneal tissue. To achieve this unmet medical need, the current banking system for corneal tissues needs to improve the reuse and recycling of these tissues through the building of a standardized workflow from lenticule donation to their distribution. Besides, novel and more efficient storage methods may be developed to maintain the biomechanical features and/or the biological activity of post-SMILE stromal lenticules. We believe that the proper preservation of lenticules is one of the main challenges for future large-scale clinical use. This narrative review has its own limitations, including the lack of a systematic search and quality assessment of included studies. The variability in study design and outcomes among the reviewed articles may contribute to heterogeneity in the results. Future systematic reviews with rigorous quality assessments are needed to validate these findings and provide more definitive conclusions.

## Data Availability

No datasets were generated or analysed during the current study.
